# Encapsulation of Capsaicin in Whey Protein and OSA-Modified Starch Using Spray-Drying: Physicochemical Properties and Its Stability

**DOI:** 10.3390/foods11040612

**Published:** 2022-02-21

**Authors:** Bo Zhang, Luyao Zheng, Siyuan Liang, Yifan Lu, Jianmei Zheng, Guoquan Zhang, Wenhao Li, Hao Jiang

**Affiliations:** College of Food Science and Engineering, Northwest A&F University, Yangling, Xianyang 712100, China; zhangbo383@163.com (B.Z.); zhengluyao130429@163.com (L.Z.); liangsiyuan21@163.com (S.L.); luyifan0222@163.com (Y.L.); zhengjm@nwsuaf.edu.cn (J.Z.); zhanggq98@126.com (G.Z.); liwenhao@nwsuaf.edu.cn (W.L.)

**Keywords:** capsaicin, encapsulation, solubility, PCA analysis, stability

## Abstract

The poor water-solubility and stability of capsaicin limits its widespread application in the industry. Spray-dried capsaicin microcapsules were fabricated using whey protein (WP) and octenyl-succinic-anhydride-modified starch (OS) as wall materials in this study. The aim is to investigate the impact of protein/starch ratio on microcapsules’ physicochemical characteristics and stability. SEM images showed that microcapsule granules were uneven in size, and irregular, with some wrinkles and dents. FTIR illustrated a chemical interaction between capsaicin and composite wall materials. XRD showed that the spray-dried powders were mainly in amorphous form. As the whey protein content decreased, the yield (9.32–68.18%), encapsulation efficiency (49.91–94.57%), wettability (158.87–232.63 s), and solubility (74.99–96.57%) of samples decreased, but the mean particle size (3.22–26.03 μm), apparent viscosity, and shear stress tended to increase. Besides, DSC revealed that the glass transition temperatures (Tg) of samples were at around 85 °C. Capsaicin microcapsules with WP:OS at the ratio of 7:3 possessed the highest Tg, and the best storage stability. Based on our research, microencapsulation significantly improved the stability and the water-solubility of capsaicin. A small amount of OSA-starch mixed with whey protein as a promising carrier for capsaicin would greatly promote the application of capsaicin in the food industry.

## 1. Introduction

As one of the most critical carotenoids, capsaicin (C_40_H_56_O_3_), with a strong spicy taste, can be obtained from ripe red pepper fruits. In addition, its healthy and valuable functions have been observed, such as anti-oxidation, anticancer, and great coloration [1]. Thus, capsaicin is extensively used in nutritious products and pharmaceutical preparations [2]. However, some less-than-ideal properties, including low water-solubility, poor stability, and pungency limit the widespread application of capsaicin.

Encapsulation is a common technique to enclose solid, liquid, and gaseous ingredients as active ingredients (core materials) using natural or synthetic macromolecular polymers (wall materials). It can protect sensitive active components from temperature, light, pH, oxygen, moisture, and metal ion to increase the shelf-life and stability [3,4]. Spray-drying is considered one of the most common microencapsulation technologies in the food industry because of its low cost, easy operation, continuous production, and excellent security [2]. In addition, the operation mechanism removes the solvent quickly when the materials to be dried are atomized in complete contact with hot air. Usually, spray-drying can transform liquids into solid particles, making it easy to incorporate and disperse in food formulations according to the prescribed dose [4].

Choosing a suitable emulsifier and wall materials is vital for encapsulation efficiency and microcapsule stability. Generally, carbohydrates (modified starch, chitosan, sucrose, and dextrin), protein (soy protein, corn protein, rice protein, collagen, whey protein, gelatin, and casein), and plant water-soluble gum (gum Arabic, carrageenan, xanthan gum, and sodium alginate) are used alone or combined with other materials as wall material in the microencapsulation of fat-soluble substances. Wang et al. [2] studied spray-dried encapsulated lutein, using gelatin and porous starch as wall materials. Bustos-Garza, Yáñez-Fernández, and Barragán-Huerta [4] used different proportions of gum Arabic, whey protein, maltodextrin, and inulin as wall materials to encapsulate astaxanthin. Lutein microcapsules were prepared by spray-drying using three components of Arabic gum, maltodextrin, and modified starch as wall materials [3]. Umaña, Turchiuli, Rosselló, and Simal [5] added a mushroom by-product enriched with polysaccharides (β-glucans) and proteins in an oil-in-water emulsion microencapsulation of sunflower oil by spray-drying. Adsare and Annapure [6] investigated structural, physicochemical, and functional properties of spray-dried powders mixed with coconut milk whey, gum Arabic (0%, 5%, 10%, or 15%), and curcumin.

Whey protein (WP) is a potential natural emulsifier due to its amphipathic structure, high nutritional value, and good film formation [7]. Jimenez, García, and Beristain [8] encapsulated conjugated linoleic acid using whey protein concentrate, and improved its stability against oxidation. Flores, Singh, and Kong [9] improved the storage properties of blueberry pomace extract after spray-drying by using whey protein isolate as the wall material. Octenyl succinic anhydride (OSA)-modified starch is synthesized by an esterification reaction, which is also a safe polysaccharide certified by the United States Food and Drug Administration (FDA), and is Generally Recognized as Safe (GRAS). It has been considered a safe emulsifier and a promising controlled release carrier material due to its amphipathic characteristics, excellent emulsion-stabilizing characteristics, and great filming properties. Recently, OSA-starch has been increasingly used as an encapsulation material to protect hydrophobic substances [10,11,12]. Furthermore, more attention has been paid to the application of protein-polysaccharide in stabilizing emulsions [13]. Protein-polysaccharide conjugates can be formed via electrostatic interaction or covalent bonds [14]. The steric hindrance effect and viscosity of polysaccharides can be used to enhance the stability of the emulsion. Zhou et al. [13] investigated the impact of soy protein isolate-dextran conjugation on capsicum oleoresin (*Capsicum annuum* L.) nanoemulsions.

However, there are rarely studies on the physicochemical characteristics and stability of capsaicin microcapsules by spray-drying using whey protein and OSA-modified starch combined as wall materials. Thus, the objective of our investigation was to: (1) microencapsulate capsaicin by spray-drying using different mass ratios of WP-to-OSA starch as wall materials; (2) evaluate the physicochemical properties of capsaicin powders and their stability; (3) select capsaicin microcapsules with better performances in different proportions, which could be used as stable water-soluble pigments and functional ingredients in clean-label food products.

## 2. Materials and Methods

### 2.1. Materials

Whey protein (purity 80%, Hefei Bomei Bio-Technology Co., Ltd., Hefei, China) was used. Capsaicin (80%, pure) was purchased from Beijing Kairiji Biotechnology Co., Ltd., Beijing, China. Potatoes (*Solanum tuberosum* L.) were selected from a local supermarket in Yangling, Shaanxi, China. Octenyl succinic anhydride (OSA, purity 99%), medium chain triglyceride (MCT), and the Nile red (purity 98%) were purchased from Shanghai Yuanye Bio-Technology Co., Ltd., Shanghai, China. In addition, DMSO, phenolphthalein, potassium bromide, anhydrous ethanol, and sodium hydroxide were of analytical grade. Potato starches were extracted by water milling, with a chemical composition of 0.32% ash, 26.37% amylose, 0.06% protein, and 0.32% fat. The degree of substitute (DS) of potato starch denatured after OSA was 0.0156.

### 2.2. Starch Modification by OSA

Potato starches were modified with OSA according to the method of He, Liu, and Zhang [15], with slight improvements. First, potato starch (35 g) mixed with 65 mL distilled water was placed into a 35 °C magnetic stirrer for 1 h. Then, 3.0% NaOH was added into the slurry drop by drop to adjust the pH to 8.4 ± 0.1. Next, 1.05 mL OSA (3% of starch weight) diluted with 5.25 mL anhydrous ethanol was added at a constant rate within 2 h. At the same time, 3.0% NaOH was added to maintain the pH within the range of 8.4–8.5. The whole reaction was held at 35 °C for a total of 3 h. Then, the pH was adjusted to 6.5 ± 0.1 by adding 0.5 mol/L HCl solution. Finally, OSA-modified starch (OS) was washed with deionized water and anhydrous ethanol 3 times, respectively, and dried, milled, and passed through a 100-mesh sieve.

### 2.3. Preparation of Capsaicin Emulsion and Microcapsules

Whey protein (WP) was dissolved in the distilled water, and stirred overnight (12 h) in a magnetic stirrer to swell fully. OS mixed with the distilled water was heated at 65 °C and stirred for 30 min, and then placed overnight at room temperature. After then, WP and OS were mixed in ratios of 10:0, 9:1, 7:3, 5:5, 3:7, 1:9, and 0:10 as the mixture of encapsulating agents (20.0 g total), and stirred for 40 min to obtain the aqueous phase. Next, 100 mg capsaicin (0.5% of the encapsulating agent weight) was dissolved in 4 g MCT at 180 °C to get the oil phase. Subsequently, it was added to the aqueous phase drop by drop, and stirred for 10 min to obtain a total of 400 g of emulsion. Finally, the oil–water mixture was homogenized (10,000 r/min, 6 min) using a high-speed dispersion homogenizer (FJ200-S, Shanghai Suoying Instruments Co., Shanghai, China), and then was treated for 5 min with an ultrasonic cell crusher (SCIENTZ-IID, Ningbo Xinzhi Biotechnology Co. Ltd., Ningbo, China; the power was 360 W; the temperature was controlled by an ice water bath below 45 °C) to obtain capsaicin emulsions.

**Figure 1:**
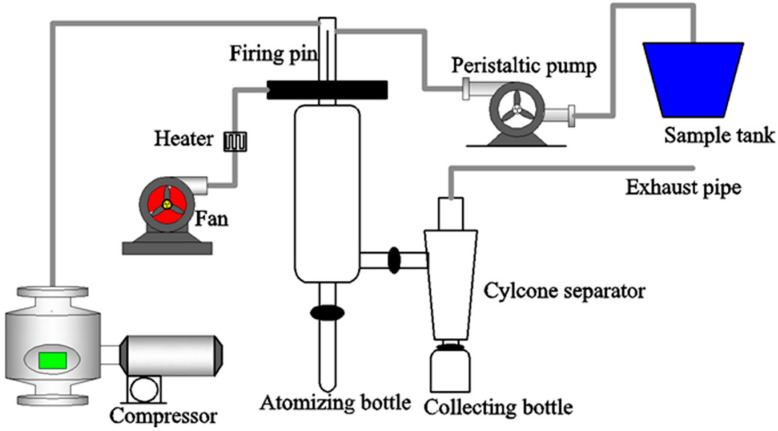
Schematic diagram of experimental spray-drying system.

The drying process was performed by an SP-1500 spray-dryer (Shanghai SunYi Tech Co., Ltd., Shanghai, China) with an inlet air temperature of 185 ± 5 °C, and outlet air temperature of 85 ± 5 °C. The schematic diagram of the experimental spray-drying system used in our study is shown in Figure 1. The emulsions were magnetically stirred during the whole drying process. The preparation process was wrapped with tin foil, and carried out in dark conditions. No microcapsule powder was obtained from the samples with the ratio of WP to OS of 0:10.

### 2.4. Light Microscopy (LM)

Emulsions were visualized by digital light microscopy (DMBA400, Motic, Beijing, China) at 400-times magnification.

### 2.5. Fluorescence Microscopy (AFM)

1 mg Nile red (with an excitation wavelength of 488 nm) was dispersed in 1 mL DMSO to prepare the fluorescent dye solution. Fresh emulsions of 0.5 mL were evenly mixed with 20 μL of fluorescent dye solution. Then, the sample (9 μL) was observed using fluorescence microscopy (LECIA DM6 B, Lecia, Weztlar, Germany) at 200-times magnification.

### 2.6. Rheological Properties

Steady shear analysis of fresh capsaicin emulsions was determined using a rheometer (DHR-1, Waters, Milford, MA, USA) equipped with a parallel aluminum plate diameter of 40 mm. Approximately 1.25 mL samples were used, and the exposed surface of the sample was covered with a thin layer of low-viscosity silicone oil to avoid water evaporation. The measurement conditions were as follows: the mode was flow scanning, the temperature was 25 °C and 50 °C, the shear rate was 0.01~100 s^−1^, and 10 points were selected for each order of magnitude. The curves were fitted using the Herschel–Bulkley model in Origin 2021, namely, τ = τ0 + Kγn, where τ (Pa) is the shear stress, τ0 (Pa) is the yield stress, K (Pa·sn) is the consistency coefficient, γ (s^−1^) is the shear rate, n is the fluid index, and R^2^ represents the degree of fit.

### 2.7. Characterization of Microcapsules

#### 2.7.1. Scanning Electron Microscopy (SEM)

The capsaicin microcapsules were fixed on the aluminum platform with conductive adhesive, then sprayed with gold, and visualized by scanning electron microscopy (Nano SEM-450, FEI, Oregon, Portland, OR, USA) at 6000× magnification.

#### 2.7.2. Fourier Transform-Infrared Spectroscopy (FT-IR)

The samples were prepared by the tableting method: the mixture of 1 mg microcapsules and 100 mg dried KBr was finely ground and pressed into tablets. The conditions were as follows: the background was the KBr sheet, the wavenumber range was 4000–400 cm^−1^, and the resolution was 4 cm^−1^. The samples were measured by a Fourier transform infrared spectrometer (Vetex70, Bruker, Karlsruhe, Germany).

#### 2.7.3. X-ray Diffraction (XRD) Pattern

The crystal properties of microcapsules were determined by a D8 Advance X-ray diffractometer of the Germany Bruker Company. The measurement conditions were: Cu-Kα target; tube voltage was 40 kV; current was 40 mA; scanning speed was 6°/min; step length was 0.02°; and measurement angle was 2θ = 4~50°.

#### 2.7.4. Yield of Encapsulation

The formula of yield (*Y*) is as follows:

$$Y\;(\%)=\;\frac{\mathrm{microcapsules}\;\mathrm{weight}\;(g)}{\mathrm{the}\;\mathrm{weight}\;\mathrm{of}\;\mathrm{non-solvent}\;\mathrm{mass}\;\mathrm{in}\;\mathrm{the}\;\mathrm{feed}\;(g)}\;\times \;100$$

#### 2.7.5. Encapsulation Efficiency of Microcapsules

The formula of encapsulation efficiency (*EE*) was measured following the method of Zhao et al. [16] with a few modifications:

$$\mathrm{EE}\;(\%)=\left(1-\frac{\mathrm{unencapsulated}\;\mathrm{capsaicin}\;}{\mathrm{total}\;\mathrm{capsaicin}}\right)\;\times \;100$$

(1). The standard curve of capsaicin

Capsaicin (50 mg) was dispersed in 500 mL absolute ethyl alcohol to obtain a 10 mg/100 mL capsaicin standard solution. The absorbance values (A) were measured at 460 nm from 1.0 mg/100 mL~10 mg/100 mL capsaicin standard solutions. The standard curve of capsaicin was drawn with the concentration (C) of capsaicin as the abscissa, and the absorbance value as the ordinate:

A = 0.0811C + 0.004 (R^2^ = 0.9999).

(2). The total capsaicin content measurement

Samples of 100 mg were dispersed entirely in 5 mL deionized water with the aid of an ultrasonic machine (KQ5200DE, Kunshan, China). Then, the dispersions were transferred into a 100 mL brown volumetric flask, and centrifuged (4000 r/min, 4 °C) for 10 min. The absorbance values were measured at 460 nm using 95% (*v/v*) ethanol solution as blank control.

(3). The unencapsulated capsaicin measurement

Capsaicin microcapsules (200 mg) were accurately weighed into a 50 mL centrifuge tube before a 20 mL absolute ethyl alcohol was added, and shook for 1 min. Then, it was centrifuged (4000 r/min, 4 °C) for 10 min. Supernatant (5 mL) was transferred into a 25 mL brown volumetric flask, and then, the absorbance values were measured at 460 nm using ethanol solution as blank control.

#### 2.7.6. The Moisture Content

The moisture content of capsaicin microcapsules was measured according to the AOAC 930.15 method [17].

#### 2.7.7. Solubility Measurement

The capsaicin microcapsule (3 g, dry basis) was dissolved in 30 mL deionized water. Then, the sample was centrifuged (4000 r/min, 4 °C) for 10 min, and the supernatant was poured out. The above steps were repeated several times, and the precipitation was washed into an aluminum box with known mass, and baked to a constant weight. The solubility (*S*) was calculated as:

$$S\;(\%)=[1-\frac{M_{2}-M_{1}}{M}]\;\times \;100$$

where *M*_2_ was the weight of the aluminum box and the precipitation, *M*_1_ was the weight of the aluminum box, and *M* was the sample weight.

#### 2.7.8. Wettability of Microcapsules

Approximately 0.1 g microcapsule powder was sprinkled in 50 mL distilled water, stirred in a 450 rpm magnetic stirrer, and timed until the powder was completely submerged.

#### 2.7.9. Color Measurement

The color of the microcapsule was measured using the colorimeter (Ci7600, Aiseli Color Technology Co., Ltd., Shanghai, China). First, the L*, a*, and b* values were recorded, and the total color difference (Δ*E*) was calculated as follows:

$$\Delta E=\sqrt{{\left(L^{*}-L_{0}^{*}\right)}^{2}+{\left(a^{*}-a_{0}^{*}\right)}^{2}+{\left(b^{*}-b_{0}^{*}\right)}^{2}}$$

where L*, a*, and b* represent the color of the microcapsule samples; *L*_0_*, *a*_0_*, and *b*_0_* represent the color of the sample with the ratio of WP to OS of 10:0.

#### 2.7.10. Thermal Properties Measurement

A differential scanning calorimeter (DSC, Q2000, Waters, Milford, MA, USA) was used to analyze the thermal property of microcapsules. The samples (3 mg) were scanned from 30 °C to 250 °C at 10 °C/min. The flow rate of nitrogen was 20 mL/min. The DSC curve was analyzed with TA software.

#### 2.7.11. Particle Size Distribution

A laser particle size analyzer (LS13320, Beckman Coulter, CA, USA) was used to measure the capsaicin microcapsules. The sample processing module was the universal liquid module ULM.

### 2.8. Stability of the Capsaicin Microcapsules

Three microcapsules with greater encapsulation efficiency were stored for 15 days at different storage temperatures (25 °C, 50 °C) and different light conditions (UV light, sunlight). Samples of 100 mg were taken out every 3 days to measure the total capsaicin content. The capsaicin retention rate (*R*) was calculated with following formula:

$$R\;(\%)=\frac{\mathrm{residual}\;\mathrm{capsaicin}\;\mathrm{content}}{\mathrm{initial}\;\mathrm{capsaicin}\;\mathrm{content}}\;\times \;100$$

### 2.9. Statistical Analysis

All measurements were performed in triplicate. The data were expressed as mean ± standard deviation. Excel 2013, Minitab 18.1, SPSS 20.0, and Origin 8.0 were used for statistical analysis (Tukey, *p* < 0.05) and plotting.

## 3. Results and Discussion

### 3.1. Morphological Characteristics of Capsaicin Emulsion

**Figure 2:**
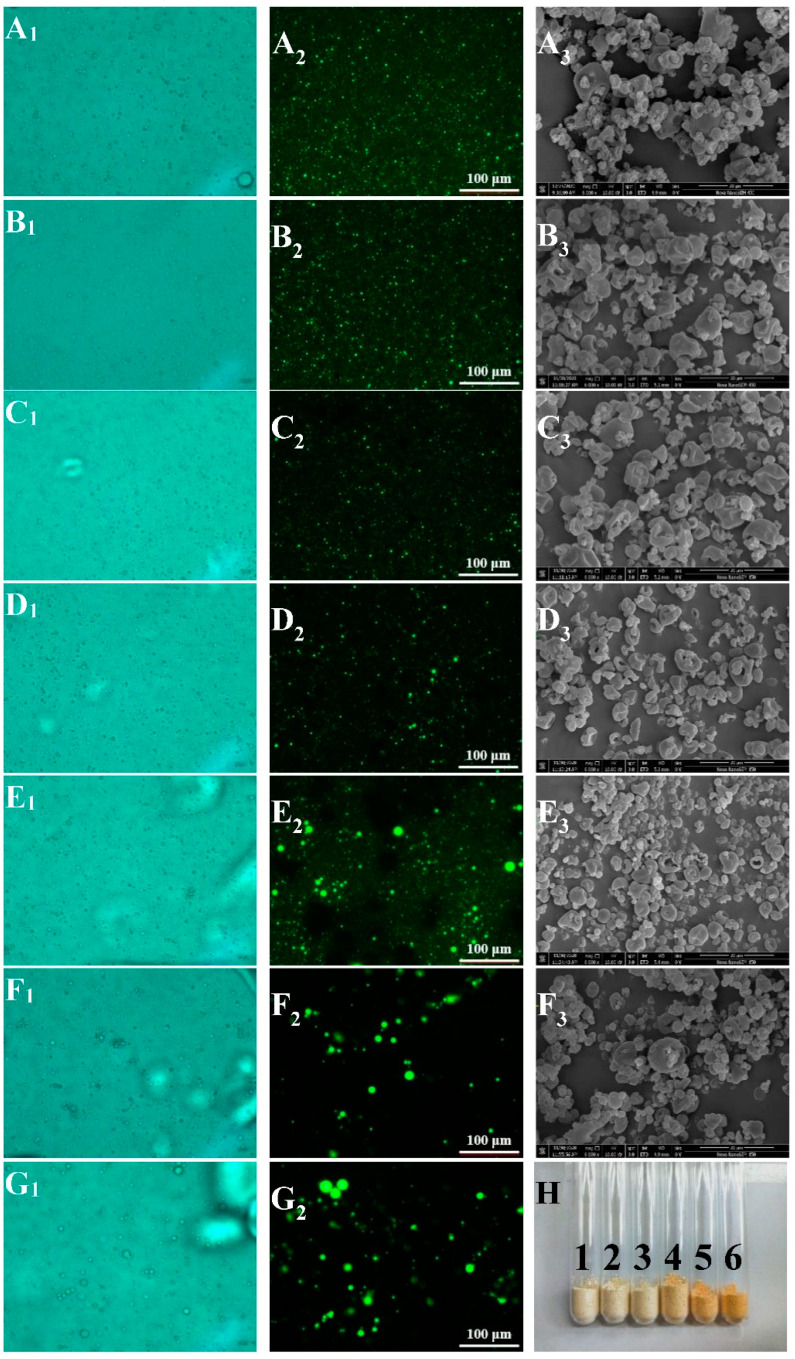
Light micrographs (×400) (1) and fluorescence micrographs (AFM) (×200) (2) of WP-OS-capsaicin emulsion, scanning electron micrographs (SEM) (×6000) (3), and appearance (**H**) of microcapsules. (**A**–**G**) represent WP (whey protein):OS (OSA-modified starch) at 10:0, 9:1, 7:3, 5:5, 3:7, 1:9, and 0:10, respectively; 1~6 in (**H**) represent WP (whey protein):OS (OSA-modified starch) at 10:0, 9:1, 7:3, 5:5, 3:7, and 1:9, respectively.

The morphological characteristics of capsaicin emulsion observed from light microscopy and fluorescence microscopy are shown in Figure 2(A_1_–G_2_). Capsaicin was dissolved in MCT, and stained with Nile red (labeled green). As shown in the LM and AFM figures, the droplets were tiny, uniform, and evenly distributed in the emulsions with WP:OS ratios of 10:0, 9:1, 7:3, and 5:5. This indicated that the capsaicin emulsions with higher WP content possessed great emulsifying capacity, and could effectively prevent the fusion between droplets. The samples with WP:OS ratios of 3:7 and 1:9 appeared to have large droplets and droplet aggregates, and extremely uneven separation of large and small droplets, suggesting that the compound emulsifier with more excellent OS in our study exhibited a poor emulsifying effect. Additionally, the emulsions only stabilized by OS presented larger droplets, which revealed severe droplet aggregation in the sample, and a poor emulsification effect of a single OS.

### 3.2. Rheological Properties of Capsaicin Emulsion

**Figure 3:**
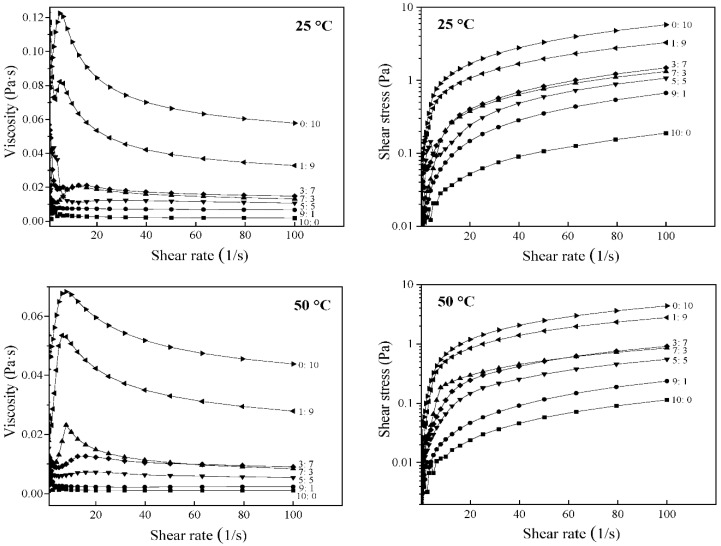
Shear rheological curve of WP-OS-capsaicin emulsion. 10:0, 9:1, 7:3, 5:5, 3:7, 1:9, and 0:10 represent WP (whey protein):OS (OSA-modified starch).

The steady flow behaviors of the WP-OS-capsaicin emulsion at 25 °C and 50 °C are shown in Figure 3. With the increase of shear rate, the apparent viscosity of the samples tended to decrease. Besides, the rheological curves exhibited different degrees of the convex shear stress axis. Thus, all samples were non-Newtonian fluids presenting pseudo-plastic and shear-thinning characteristics [18]. Under the same temperature, the apparent viscosity and shear stress tended to decrease with the increasing whey protein content in the emulsifier. This might be owing to the interaction between whey protein and OSA-denatured starch homogenizing, and the ultrasonic treatment [19]. Also, the system with excellent apparent viscosity may be attributed to the formation of strong and continuous networks.

The fitting parameters of the Herschel–Bulkley equation for steady flow tests are listed in Table 1. The coefficient of determination (R^2^) of all curves was more significant than 0.99, which indicated that the Herschel–Bulkley equation fitted well with the data. The flow index (n) of all samples was less than 1, except the sample with WP:OS as 9:1 at 50 °C, which exhibited non-Newtonian pseudoplastic behavior. The addition of OS increased the system’s consistency coefficients (K), indicating that the strong and dense network structure responsible for the thickening effect was formed [19]. In addition, the values of n in the system at 25 °C were lower than that at 50 °C, whereas the values of K at 25 °C were more remarkable than that at 50 °C (Table 1). This illustrated that the temperature could affect the flow index and the consistency coefficient of the emulsion. The strong bond between whey protein and OSA-starch at a lower temperature might promote the stability of the emulsion system, and reduce the degree of tensile deformation under the external action, resulting in a higher K value, and a lower n value.

### 3.3. SEM and Appearance Analysis

Figure 2(A_3_–F_3_) and H show the SEM and appearance images of capsaicin microcapsules. Capsaicin powders were fine, fluffy, had no accumulation, and presented uniform color and dispersion (Figure 2H), illustrating the powder products formed by rapid water evaporation after spray-drying were of good quality. Figure 2(A_3_–F_3_) shows that the boundaries between microcapsule granules were distinct, and there was no adhesion phenomenon. The membrane structure on the surface was dense and continuous, but the particle size was not remarkably uniform. In addition, wrinkles and dents could be seen around some microcapsule particles.

This was because the nozzle atomizes the emulsion in the drying process, and the contact area between the droplets and the hot air is significantly increased. As a result, water evaporates rapidly, resulting in the shrinkage of the film-forming materials, which is a common phenomenon in preparing powdered substances by spray-drying [20]. In addition, the crystal structure of the microcapsule is damaged in the late drying period, and the lack of strong support of the crystal structure will also lead to the collapse of the particles from all sides to the inside, resulting in the sagging phenomenon.

### 3.4. FT-IR Spectroscopy Analysis

**Figure 4:**
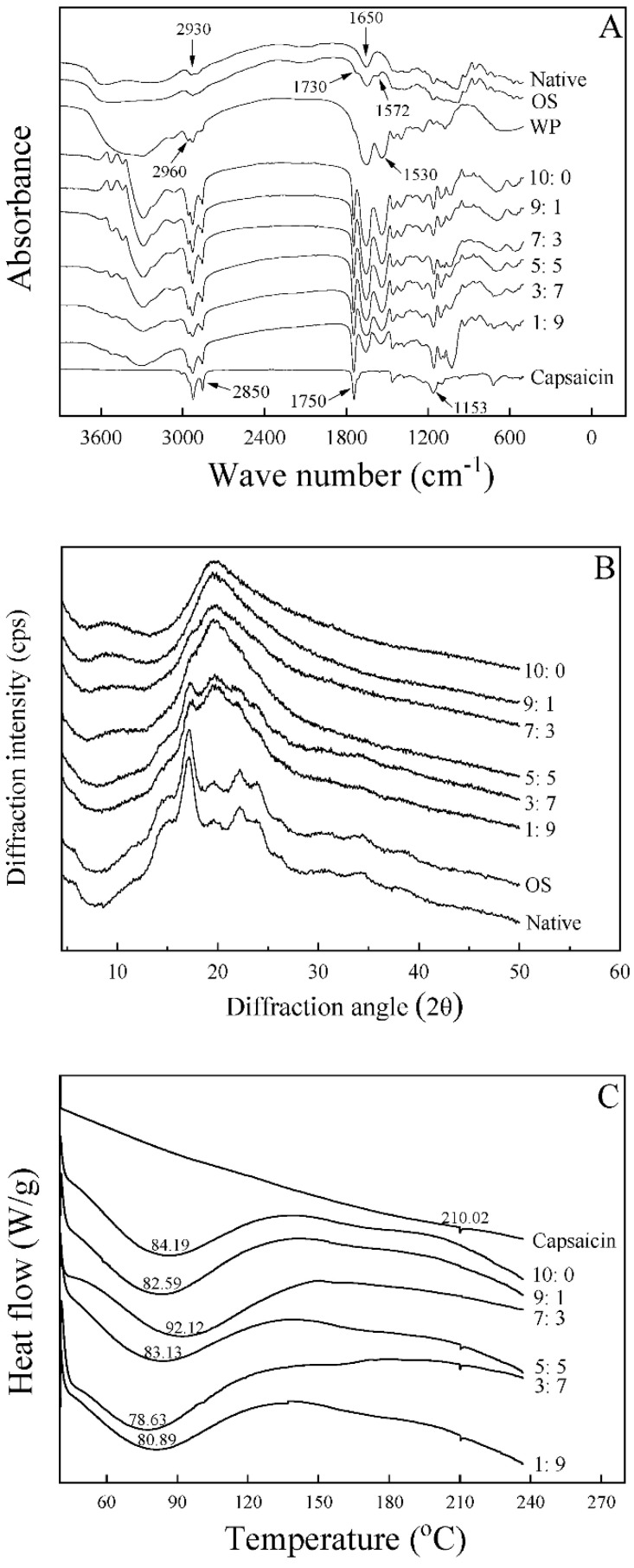
FTIR spectra (**A**), X-ray diffraction pattern (**B**), and DSC thermograms (**C**) of WP-OS-capsaicin microcapsules. Native, native starch; 10:0, 9:1, 7:3, 5:5, 3:7, and 1:9 represent WP (whey protein):OS (OSA-modified starch).

The FT-IR spectra of native starch, OS, capsaicin, and microcapsules were shown in Figure 4A. Native starch and OS exhibited similar FTIR spectra patterns, with characteristic absorbance peaks at 3200–3600, 2930, and 1650 cm^−1^. Two new absorption bands at 1730 cm^−1^ (C=O) and 1572 cm^−1^ (RCOO-) appeared in OS curves concerning native potato starch. It was proven that new groups were introduced into starch through esterification. In addition, four small peaks at 2930 cm^−1^, 2850 cm^−1^, 1750 cm^−1^, and 1153 cm^−1^ were all the characteristic absorption peaks of pure capsaicin. The characteristic absorbance peak of the encapsulated capsaicin powders corresponded to wall materials. Furthermore, the absorption peaks of microcapsules at 1750 cm^−1^ (C=O) and 1153 cm^−1^ (C–O) were more substantial, longer, and narrower than the peak of pure capsaicin, which was related to the embedding of capsaicin in the microcapsules, indicating that the capsaicin of the core materials had a strong binding effect with the wall materials to form a stable microcapsule system. Therefore, it was deduced that a chemical interaction existed between capsaicin and the wall materials in spray-dried powders.

### 3.5. X-ray Diffraction Patterns

The XRD spectra of the spray-dried capsaicin microcapsules are illustrated in Figure 4B. The samples obtained with WP:OS ratios of 1:9 and 3:7 presented intense diffraction peaks at 17.3°, 19.7°, and 22.2°, and weak diffraction peaks at 15° and 24° (Figure 4B). Along with the decrease of OS in microcapsules, specific peaks progressively disappeared. The microcapsules with WP:OS as 5:5 and 7:3 showed a typical rise at 20.0°, and there existed two diffraction peaks at 9.5° and 17.3°. The intense peak presented at 20.0° for microcapsules obtained with WP:OS ratios of 9:1 and 10:0, and there was weak diffraction at 17.3°. The microcapsules with more excellent WP gradually tended to have an intense diffraction peak only at 20.0°, indicating the microcapsules were mainly in amorphous form. A higher water-solubility typically characterizes the amorphous state [21].

### 3.6. Yield, Encapsulation Efficiency, Moisture, Wettability, and Solubility

Characterization of the microcapsules is considered essential for the food industry. Table 2 summarizes the yield parameters, encapsulation efficiency, moisture, wettability, and solubility of the samples. The yield of capsaicin microcapsules was between 9.32% and 68.18%. Increases in OSA-modified starch led to decreases in the yield process, which could be due to the higher viscosity of the mixture, causing stickiness in the dry chamber wall [22]. Nevertheless, the result was consistent with the development in rheological properties.

The encapsulation efficiency (EE) ranged from 49.91% to 94.57%, where it was shown that the samples with WP and OS at weight ratios of 10:0, 9:1, and 7:3 processed no noticeable difference, whereas the three microcapsules presented the higher EL among the other samples. Thus, the capsaicin microcapsules with good EL indicated the wall materials had excellent film-forming capability, and WP and OS with ratios of 10:0, 9:1, and 7:3 presented a good interaction [3]. In addition, capsaicin microcapsules with higher modified starch in the composition achieved lower EE, suggesting that the modified starch did not show an excellent capability of film-formation properties.

The moisture of all microcapsules samples varied between 0.33% and 3.00%, except the sample obtained with a WP:OS ratio of 3:7, which presented low moisture (<4%, the minimum specification for powders used in the food industry). Low humidity in microcapsules can prevent mildew degradation and moisture absorption, and agglomeration reduces the dispersion of active ingredients, and avoids microbial growth, improving the physical and chemical stability [23,24].

The wettability of powders presents the ability to absorb water connected with the reconstitution of the microcapsules [24]. The wettability of capsaicin microcapsules was between 158.87 and 232.63 s. The sample obtained with a WP:OS ratio of 10:0 displayed a good wettability (158.87 s), whereas the sample with a WP:OS ratio of 1:9 presented a poor wettability (232.63 s). It was reported that the wettability was related to the particle size in the system [25]. A smaller granule size increased the surface area of microcapsules that improve the interaction with water. Similar results were reported by Chew, Tan, and Nyam [24] for the microencapsulation of refined kenaf seed oil by spray-drying.

Solubility is vitally important in food product processing [26]. The solubility oscillated from 74.99% to 96.57% in this study (Table 2). Along with the increase in the content of esterified starch, the solubility gradually decreased. Thus, free capsaicin could not be dissolved in water. In contrast, the capsaicin microcapsule was relatively soluble, indicating encapsulated capsaicin with WP and OS as wall materials after spray-drying could improve the solubility. This could be consistent with the result of the XRD analysis.

### 3.7. Color Analysis

The results of the color measurement of capsaicin microcapsules are shown in Table 3. The L* value of the microcapsules obtained with WP:OS ratios of 10:0, 9:1, and 7:3 showed no significant difference, though they were greater than other samples (*p* < 0.05). The samples with higher L* indicated that there was less capsaicin exposed on the surface of the microcapsules, thus improving the microencapsulation effect, which was consistent with the result of encapsulation efficiency.

There was no significant difference in a* between the samples with WP and OS at weight ratios of 10:0, 9:1 and 7:3, whereas the three kinds of microcapsules achieved lower a* values among other samples. Both b* and Δ*E* had the same trends as the value of a*. Besides, along with the increase in the content of OS, values of a*, b*, and Δ*E* gradually increased. This indicated that the color of capsaicin microcapsules in our investigation tended to be red and yellow with the increase of the content of OS.

### 3.8. Thermal Properties

DSC thermograms of the capsaicin and capsaicin microcapsules are given in Figure 4C. The DSC curve of native capsaicin showed a sharp peak at 210.02 °C, which was the melting point of the capsaicin crystal. Fast evaporation in spray-drying produced the microcapsules in the amorphous form, which presented the glass transition [27,28]. The results of XRD above are consistent with this conclusion. Figure 4C exhibits the glass transition temperatures (Tg) of microcapsules with WP:OS ratios of 10:0, 9:1, 7:3, 5:5, 3:7, and 1:9, which were 84.19, 82.59, 92.12, 83.13, 78.63, and 80.89 °C, respectively. The sample with a WP:OS ratio of 7:3 processed the highest Tg compared to the other five powders, indicating that a higher heat was required for a phase transition, and the microcapsule (WP:OS = 7:3) had a relatively high thermal stability. In addition, the Tg of the six products was higher than the room temperature, which revealed that samples could maintain a stable glass state at room temperature [29].

### 3.9. Stability of the Capsaicin Microcapsules

**Figure 5:**
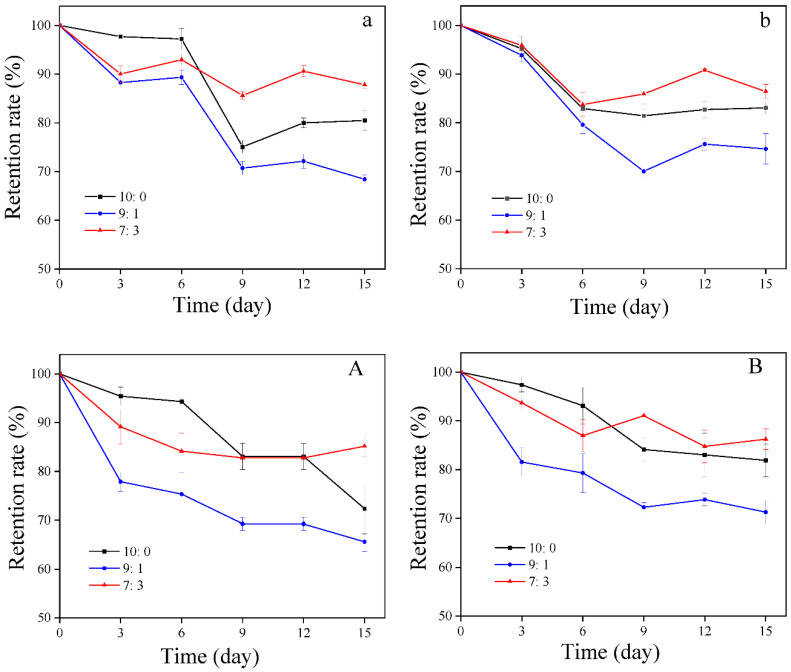
Retention rate of WP-OS-capsaicin microcapsules under different storage conditions ((**a**), 50 °C in the dark; (**b**), 25 °C in the dark; (**A**), 25 °C in the UV light; (**B**), 25 °C in the lightness). 10:0, 9:1, and 7:3 represent WP (whey protein):OS (OSA-modified starch). Error bars represent standard deviation.

Microcapsules with excellent stability can significantly increase the chances of their application in industrial processing [30]. Therefore, stability is an important index to evaluate the quality of microcapsules. Figure 5 shows the retention rate of capsaicin in three microcapsules with relatively greater encapsulation efficiency under different storage conditions. It was found that the capsaicin retention rate in the microcapsules with WP:OS ratios of 7:3 was 87.81% at 50 °C and 86.41% at 25 °C on the fifteenth day. However, its retention rate in microcapsules with WP:OS ratios of 10:0 and 9:1 were below 85% (80.47% for the sample with a WP:OS ratio of 10:0 at 50 °C, and 83.06% at 25 °C; 68.41% for the powder with a WP:OS ratio of 9:1 at 50 °C, and 74.64% at 25 °C) on the fifteenth day (Figure 5a,b). This illustrates that the capsaicin microcapsule (WP:OS = 7:3) can more effectively improve the thermal stability of capsaicin compared to other kinds. The possible reason for this is that adding a small amount of OSA-starch promoted the WP-OS complex formation with a more stable rigid structure, which was not easy to collapse under the influence of temperature, and had a better protective effect on the core material [6].

Figure 5A,B reveal that the retention rate of capsaicin in the products with WP and OS at weight ratios of 10:0, 9:1, and 7:3 were 72.35%, 65.62%, 85.15% under UV light; and 81.89%, 71.26%, 86.25% under lightness on the fifteenth day, respectively. Microencapsulation and possible hydrogen bonding with WP-OS complexes may offer protection to capsaicin under light conditions, and prolong shelf-life during storage [31]. The retention rate of capsaicin microcapsules with a WP:OS ratio of 7:3 was similar under UV light and lightness after fifteen days. The effective protection of capsaicin from degradation at this ratio may result in no significant difference between the two groups over a short period of time. Besides, the WP:OS ratio of 7:3 group possessed lower retention than the WP:OS ratio of 10:1 group during the initial six days. The lower retention rate may be related to the higher capsaicin content on the sample surface.

### 3.10. Principal Component Analysis

**Figure 6:**
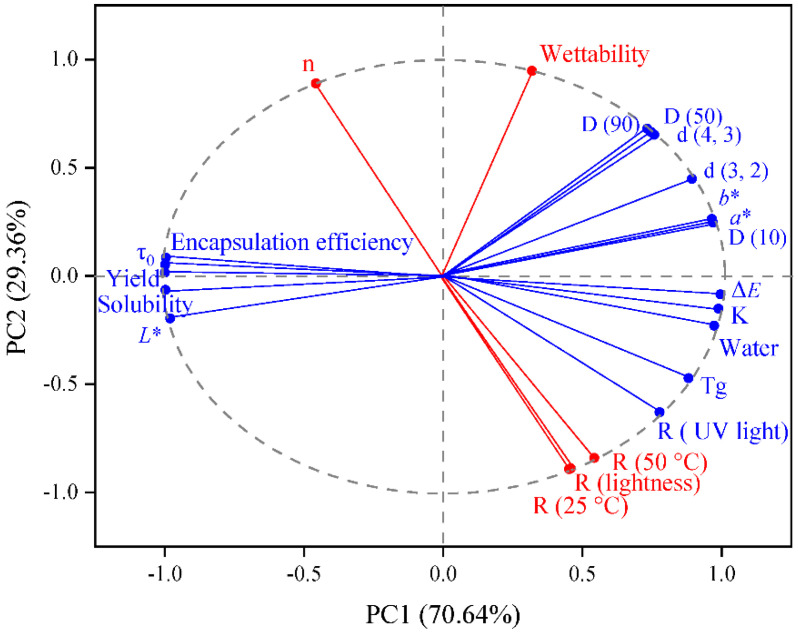
Principal component analysis of group differences.

Group differences (yield, encapsulation efficiency, water, wettability, solubility, color, particle size distributions, Tg, shear rheological property, and stability) were determined using a principal component analysis (PCA). As illustrated in Figure 6, none of the variables are correlated with any PCs directly. The yield mainly influences PC1 (70.64%), encapsulation efficiency, water, solubility, color, particle size distributions, Tg, τ0, and K. Besides, wettability, n, and stability contribute to the construction of PC2 (29.36%). This suggested that most components were interrelated, and affected the physicochemical properties of microcapsules together. The results of mathematical analysis using PCA were consistent with the theoretical analysis in our investigation.

## 4. Conclusions

Capsaicin microcapsules possessed high water-solubility and stability. It was verified that the emulsifying capacity, yield, encapsulation efficiency, solubility, and L* decreased as OSA-starch concentration increased. Still, the apparent viscosity, a*, b*, Δ*E*, and mean particle size tended to increase. Capsaicin was encapsulated in microcapsules by a chemical interaction of composite wall materials in amorphous form, confirmed by FTIR and XRD. Among spray-dried capsaicin powders, the microcapsule with a WP:OS ratio of 7:3 had an excellent encapsulation efficiency and better abilities against light and heat. Hence, the complex of whey protein and a small amount of OSA-starch would be a great candidate to protect labile fat-soluble active substances. This study could also help promote capsaicin as a stable water-soluble pigment and functional ingredient in the clean-label food industry.

## Figures and Tables

**Table 1:** Fitting parameters of Herschel–Bulkley equation for WP-OS-capsaicin emulsion ^a,b^.

Temperatures	WP:OS	τ0 (Pa)	K (Pa·s^−1^)	n	R^2^
25 °C	10:0	0.0062 ± 0.0001 ab	0.0034 ± 0.0001 f	0.8667 ± 0.0053 c	0.9965
	9:1	0.0021 ± 0.0003 b	0.0084 ± 0.0000 e	0.9518 ± 0.0006 a	0.9998
	7:3	−0.0081 ± 0.0014 c	0.0294 ± 0.0027 c	0.8320 ± 0.0202 d	0.9968
	5:5	0.0126 ± 0.0008 a	0.0163 ± 0.0001 d	0.9077 ± 0.0021 b	0.9909
	3:7	0.0071 ± 0.0008 ab	0.0289 ± 0.0002 c	0.8548 ± 0.0019 cd	0.9989
	1:9	0.0025 ± 0.0000 b	0.1276 ± 0.0001 b	0.7039 ± 0.0010 f	0.9987
	0:10	−0.0306 ± 0.0048 d	0.1771 ± 0.0006 a	0.7559 ± 0.0005 e	0.9991
50 °C	10:0	0.0018 ± 0.0006 ab	0.0012 ± 0.0001 f	0.9852 ± 0.0188 b	0.9930
	9:1	0.0036 ± 0.0000 a	0.0018 ± 0.0000 f	1.0531 ± 0.0016 a	0.9981
	7:3	−0.0073 ± 0.0012 b	0.0384 ± 0.0028 c	0.6753 ± 0.0178 f	0.9930
	5:5	0.0007 ± 0.0005 ab	0.0097 ± 0.0007 e	0.8809 ± 0.0163 c	0.9978
	3:7	−0.0023 ± 0.0002 ab	0.0172 ± 0.0000 d	0.8650 ± 0.0007 c	0.9977
	1:9	−0.0336 ± 0.0066 d	0.0903 ± 0.0024 b	0.7485 ± 0.0055 e	0.9978
	0:10	−0.0230 ± 0.0006 c	0.1026 ± 0.0014 a	0.8168 ± 0.0032 d	0.9993

^a^ Different letters within the same column indicate a significant difference (*p* < 0.05), all test data are expressed as mean ± standard deviation, and the test is repeated three times. ^b^ WP, whey protein; OS, OSA-modified starch; τ0, yield stress; K, consistency coefficient; n, flow index; R^2^, coefficient of determination.

**Table 2:** Yield, encapsulation efficiency, water, wettability, and solubility of WP-OS-capsaicin microcapsules ^a,b^.

WP:OS	Yield (%)	Encapsulation Efficiency (%)	Water (%)	Wettability (s)	Solubility (%)
10:0	68.18 ± 0.51 a	94.57 ± 0.64 a	0.33 ± 0.05 c	158.87 ± 3.31 d	96.57 ± 0.14 a
9:1	64.26 ± 0.05 b	93.51 ± 0.79 a	0.48 ± 0.02 c	187.31 ± 1.60 b	92.35 ± 0.05 b
7:3	55.51 ± 0.63 c	90.43 ± 0.89 a	1.67 ± 0.56 bc	172.86 ± 1.68 c	85.77 ± 1.14 c
5:5	48.39 ± 0.34 d	80.85 ± 0.39 b	1.30 ± 0.48 c	168.33 ± 3.54 cd	80.06 ± 0.62 d
3:7	38.47 ± 0.49 e	74.37 ± 2.05 c	5.35 ± 0.14 a	195.63 ± 0.98 b	77.50 ± 0.01 e
1:9	9.32 ± 0.50 f	49.91 ± 2.58 d	3.00 ± 0.46 b	232.63 ± 2.68 a	74.99 ± 0.62 f

^a^ Different letters within the same column indicate a significant difference (*p* < 0.05), all test data are expressed as mean ± standard deviation, and the test is repeated three times. ^b^ WP, whey protein; OS, OSA-modified starch.

**Table 3:** The color, mean particle size, and size distribution of WP-OS-capsaicin microcapsules ^a,b^.

WP:OS	The Color Parameters				d (4, 3) (μm)	d (3, 2) (μm)	Particle Size Distributions (μm)		
	L* (%)	a* (%)	b* (%)	Δ*E*			D (10)	D (50)	D (90)
10:0	93.78 ± 0.39 a	2.13 ± 0.16 d	15.96 ± 0.81 d	0.59 ± 0.08 d	3.22 ± 0.21 c	1.49 ± 0.04 e	0.76 ± 0.01 f	1.78 ± 0.06 c	8.13 ± 2.07 c
9:1	93.41 ± 0.22 a	2.52 ± 0.09 d	17.27 ± 0.57 d	0.98 ± 0.56 d	24.53 ± 0.35 b	3.95 ± 0.07 d	1.24 ± 0.03 e	23.87 ± 0.58 a	51.57 ± 0.72 a
7:3	93.05 ± 0.60 a	2.83 ± 0.33 d	18.27 ± 1.45 d	2.11 ± 1.53 d	24.60 ± 0.36 b	4.79 ± 0.20 c	1.63 ± 0.04 d	23.53 ± 0.61 a	49.83 ± 0.23 ab
5:5	89.85 ± 0.23 b	8.00 ± 0.11 c	24.44 ± 0.61 c	10.63 ± 0.57 c	24.20 ± 0.36 b	5.82 ± 0.05 b	2.35 ± 0.03 c	22.37 ± 0.50 b	48.17 ± 0.47 b
3:7	87.71 ± 0.10 c	10.76 ± 0.12 b	29.07 ± 0.12 b	16.42 ± 0.18 b	24.70 ± 0.17 b	7.94 ± 0.33 a	3.67 ± 0.06 b	22.90 ± 0.17 ab	47.40 ± 0.35 b
1:9	86.60 ± 0.43 d	12.48 ± 0.50 a	32.84 ± 0.95 a	20.65 ± 1.14 a	26.03 ± 0.23 a	8.16 ± 0.25 a	6.72 ± 0.06 a	23.97 ± 0.21 a	48.40 ± 0.61 b

^a^ Different letters within the same column indicate a significant difference (*p* < 0.05), all test data are expressed as mean ± standard deviation, and the test is repeated three times. ^b^ WP, whey protein; OS, OSA-modified starch.

## Data Availability

The data presented in this study are available on request from the corresponding author.
